# Psychiatric inconsistencies of technical epidemiological nexus codes used by the Brazilian Social Service to classify work-related disorders

**DOI:** 10.47626/1679-4435-2020-597

**Published:** 2021-02-11

**Authors:** Paola Figueiredo Mylla Todeschini Alves

**Affiliations:** Private practice, Psychiatry, Curitiba, PR, Brazil

**Keywords:** psychiatric, epidemiological nexus, Brazilian law, NTEP

## Abstract

This article aims to provoke discussions about technical inconsistencies in the technical epidemiological nexus framework used as part of Brazil’s social security regulations.

Since 2007, medical experts working for Brazil’s social security administration, the INSS (Instituto Nacional do Seguro Social), apply a presumption of a causal link between sicknesses claimed by workers and the type of work they do or did, as classified by the country’s National Economic Activities Classification (CNAE- Classificação Nacional de Atividades Econômicas). Each admissible causal link, which is known as a technical epidemiological nexus (NTEP - nexo técnico epidemiológico), is defined in list C of Appendix II of Decree-Law nº 3.048/1999.^1^ The psychiatric disorders included in list C are specifically those that fall within the range F10-F48 of the International Classification of Diseases -10 (ICD-10) codes. This range encompasses mental disorders due to psychoactive substance use (ICD-10 F10-F19), psychotic disorders (ICD-10 F20-F29), depressive disorders (ICD-10 F30-F39), and anxiety and stress-related disorders (ICD-10 F40-F48). Analyzing the occupational activities that are listed as related to these disorders, ignoring the methodological biases of the study from which the associations claimed originate, certain peculiar aspects jump out.

To illustrate, take the hypothetical case of a nursing professional who works at a chemotherapy service and manifests depressive symptoms. The CNAE code for nursing is 5650 - which is not listed for any of the groups of psychiatric disorders above. Should it therefore be assumed that there is no technical epidemiological nexus between the depression manifested and the nurse’s work at the chemotherapy service? If the NTEP metric is used, ignoring any individual assessment of the case, the answer is that it depends.

If the chemotherapy service is within a hospital, the relevant CNAE would be 8610 (hospital care activities)*.* On list C, hospital activities do have a presumptive causal nexus with depressive disorders (ICD-10 F30-F39). However, if the chemotherapy service is in a clinic, rather than a hospital setting, it would be coded as CNAE 8640-2/10, which is not linked to any of the activities which it is presumed can cause depression. The work performed by the nurse is exactly the same in both hypotheses. There is no technical justification whatsoever to explain why there should be a presumed causal nexus with depression when a chemotherapy service is in a hospital, but not when it is in a different setting.

A nurse working in a chemotherapy department was used as an example, but the same reasoning applies to all health professionals who work in chemotherapy, radiotherapy, and dialysis. Their healthcare activities, individually, do not have a presumed causal nexus with depression, unless they are performed in a hospital setting. As a result, the NTEP, which should be evaluating the causal nexus between occupational activity and disease, is, in these cases, making an exclusive association with the setting in which the activity is performed - which has no relation to the reason for which it was supposedly implemented.

Another unexpected reflection of the inconsistency of NTEP data is related to professionals who manufacture items of apparel from woven and knitted fabrics. The CNAE code for this occupation is 142. In list C, CNAE 1421, which refers to these workers who specifically produce hosiery, has a presumed causal nexus with the range of codes for anxiety and stress-related disorders (ICD-10 F40-F49). There is no such presumed nexus with depressive disorder codes. The inconsistency here lies in the fact that CNAE code 1422 (manufacture of items of apparel from woven and knitted fabrics, except hosiery) is linked to the range of codes for depression, but not to anxiety. Therefore, according to the NTEP, working in the textile industry producing hosiery can cause anxiety and stress-related disorders, whereas working in the textile industry producing all other items of apparel (except hosiery) can cause depression. This raises a practical question: what if, a given clothing manufacturer produces all types of products, including hosiery? According to the NTEP rationale, workers who only produce hosiery would only be subject to developing symptoms of anxiety related to their occupation, whereas all the other workers would be prone to depression.

It is of note that in this case the underlying reasoning is the opposite of that applied to the healthcare professionals. In this example, workers performing their occupations in the same setting could develop different psychiatric symptoms; with the only variable differentiating them being whether or not they make hosiery. All of these statements can be easily verified by consulting the list in question - and one of the motives for publication of this article is to encourage readers to peruse it.

The CNAE code 8423 covers administrative occupations related to the justice department (administration and function of the judicial system and the courts, in general). The C list links this CNAE code to all of the psychiatric disorder codes, i.e., codes from ICD-10 F10 to ICD-10 F48. In turn, administrative occupations within the Federal, State, and Municipal police (CNAE 8424) are linked to almost all of the same psychiatric disorders as occupations working for the justice department (except for psychotic conditions, ICD-10 F20-F29) and also arthritis and arthrosis (ICD-10 M00-M25). What is the NTEP claiming in this case? That working in an administrative post for the police does not cause psychotic conditions, although it can cause countless other mental diseases, arthritis, and arthrosis, whereas working in an administrative post for the justice department can cause all of the psychiatric disorders covered by the C list, but does not cause arthritis or arthrosis. Following the same unpredictability as the previous examples, in this case, these are administrative occupations in the public sector, in which there is no indication, technically, of data that would explain the different diseases presumably caused by these occupations.

One occupation that merits mention is linked to the greatest number of diseases on the C list: manufacture of underwear and nightwear (CNAE 1411). With no explanation of the reasons behind these conclusions, making pajamas and underwear has a presumed causal nexus with 21 ICD-10 code ranges in the NTEP list, including tuberculosis, mental disorders, and neurological, cardiac, vascular, dermatological, and orthopedic diseases.

These brief reflections on the weak relationships in the NTEP are intended to provoke discussion of the true applicability of this type nexus. Investigation of the causal nexus between a disease and an occupation should be conducted on a case-by-case basis by a medical expert, which is still the gold standard method. The argument that this list protects workers from under-notification of work-related diseases loses its power when other data are evaluated, at least where the list of psychiatric diseases is concerned.

CNAE code 4930 (road haulage) is not linked to any of the psychiatric disorder code ranges. The NTEP presumes causal nexuses between the occupation of truck driving and diabetes, epilepsy, visual impairment, heart attack, cardiac diseases, all types of traumatism, and orthopedic sequelae. However, the major health problem faced by this class of workers is substance use and is directly related to the occupation to the extent that truck drivers consume amphetamine-based drugs to stay awake and keep working for the number of hours necessary to reach the end of their routes. The Federal government itself acknowledged that this problem is a priority when it passed Law 13.103/2015.^2^ In addition to dealing with concern about the hours they need to work (the fundamental core of the discussion, since it is the trigger for substance use), the wording of the law explicitly guarantees treatment on the state healthcare system for professional drivers who are dependent on psychoactive substance and even contains provisions allowing for outsourcing of this obligation to private institutions.

What happens in practice is that truck drivers begin to take these substances to stay awake and arrive at their destinations as quickly as possible, often after colleagues have recommended them. Many drivers will abuse these substances and may become dependent, which causes countless road accidents. In this example, substance use is caused by the occupation, but the NTEP does not cover it or ensure that this well-known problem is under-notified. It must be remembered that causing an accident, which will very often have fatal victims, can be a cause of posttraumatic stress disorder (ICD-10 code F43.2), which is another disorder that is presumed not to be caused by being a truck driver, according to the NTEP.

In view of these alarming data, it can be concluded that the NTEP does not reduce under-notification of many work-related diseases. On the contrary, it helps to mask the true cause of some of them. Furthermore, it casts doubt on medical conclusions because of the lax and disconnected
